# A case study on the potential angiogenic effect of human chorionic gonadotropin hormone in rapid progression and spontaneous regression of metastatic renal cell carcinoma during pregnancy and after surgical abortion

**DOI:** 10.1186/s12885-015-2031-1

**Published:** 2015-12-24

**Authors:** László Mangel, Krisztina Bíró, István Battyáni, Péter Göcze, Tamás Tornóczky, Endre Kálmán

**Affiliations:** 1 Institute of Oncotherapy, University of Pécs, H-7624, Édesanyák útja 17, Pécs, Hungary; 2 Department of Chemotherapy, National Institute of Oncology, Budapest, Hungary; 3 Department of Radiology, University of Pécs, Pécs, Hungary; 4 Clinic of Obstetrics and Gynecology, University of Pécs, Pécs, Hungary; 5 Institute of Pathology, University of Pécs, Pécs, Hungary

**Keywords:** Human chorionic gonadotropin hormone, Luteinizing hormone receptor, Metastasis, Pregnancy, Renal cell carcinoma

## Abstract

**Background:**

Treatment possibilities of metastatic renal cell carcinoma (mRCC) have recently changed dramatically prolonging the overall survival of the patients. This kind of development brings new challenges for the care of mRCC.

**Case presentation:**

A 22 year-old female patient with translocation type mRCC, who previously had been treated for nearly 5 years, became pregnant during the treatment break period. Follow-up examinations revealed a dramatic clinical and radiological progression of mRCC in a few weeks therefore the pregnancy was terminated. A few days after surgical abortion, CT examination showed a significant spontaneous regression of the pulmonary metastases, and the volume of the largest manifestation decreased from ca. 30 to 3.5 cm^3^ in a week. To understand the possible mechanism of this spectacular regression, estrogen, progesterone and luteinizing hormone receptors (ER, PGR and LHR, respectively) immuno-histochemistry assays were performed on the original surgery samples. Immuno-histochemistry showed negative ER, PGR and positive LHR status suggesting the possible angiogenic effect of human chorionic gonadotropin hormone (hCG) in the background.

**Conclusion:**

We hypothesize that pregnancy may play a causal role in the progression of mRCC via the excess amount of hCG, however, more data are necessary to validate the present notions and the predictive role of LHR overexpression.

## Background

Treatment possibilities of metastatic renal cell carcinoma (mRCC) have changed dramatically in the last decade from conventional cytokine-based chemo-immunotherapies to therapies using broad spectra of targeted drugs and, most recently, immune system modulator agents [1–3]. These new treatment modalities have increased the median overall survival of mRCC beyond two years, naturally poor, moderate and good risk patients still have different clinical outcomes [1–4]. As a consequence of this kind of development and the increasing number of fertile female patients surviving for a long time it has become more important to get acquainted with the possible interaction of the progression of mRCC with pregnancy and child-bearing potential.

Both treatment possibilities and the outcome of cancer diseases during pregnancy are well discussed in the medical literature [5–14]. Numerous publications report successful pregnancies and deliveries in the case of breast cancer, gynecological tumors and hematological malignancies, with the respect of the oncologic point of view. In some tumor entities (e.g., breast cancer over the first trimester, early kidney tumors, etc.) the treatment recommendations are similar to those for non-pregnant women, in other cases the treatment decisions have to be considered under critical evaluation [5–14]. Several case reports and reviews are also available concerning the challenges associated with kidney cancers diagnosed in pregnant women [15–22]. However, there is only limited knowledge about the behavior of metastatic renal cell carcinoma during pregnancy so far [23, 24].

Here we review the case of a very young female patient with disseminated kidney cancer who became pregnant after her initial anticancer treatment. Her disease progressed quickly therefore surgical abortion had to be carried out. Following the abortion an amazing clinical and radiological improvement was observed without any further therapeutic intervention. The rate and extent of the tumor regression was more outstanding than it could have been expected due to any kind of effective anticancer treatment.

## Case presentation

**Fig. 1:**
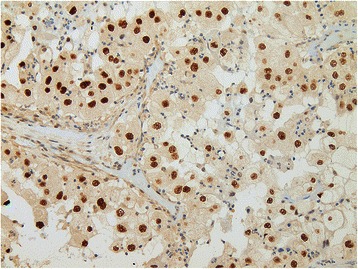
Xp 11.2 translocation carcinoma, with TFE3 fusion protein immunostaining

The primary check up of a 16-year-old, twin-born female Caucasian patient with no relevant medical history started due to both weight loss and a mass which was found in the right kidney. Nephrectomy was carried out in January 2007. Macroscopically a 14 cm large, solid and cystic tumor mass was seen with focal necroses and haemorrhages (pT2 pN0). Histology showed a juvenile Xp 11.2 translocation type renal cell carcinoma (Fig. 1). The tumor cells were arranged in papillary or trabecular-alveolar structures. They had large, clear to light pink cytoplasm and small nucleoli. Just a few mitoses were seen and no vascular invasion was detected. The immuno-histochemical (IHC) analysis proved focal EMA, CK (AE1-3) and CD10 reactions. The TFE-3 staining showed intense nuclear reaction.

The patient was only observed till August 2007, when an intraperitoneal relapse was confirmed. Following metastasectomy chemo-immunotherapy was initiated with the combination of recombinant interferon alfa 2a and vinblastine. In February 2008 sunitinib therapy was introduced due to local, peritoneal and pulmonary progression. Continuous regression was observed until March 2009, when mediastinal-hilar relapse was revealed. The dose of sunitinib was increased from a daily 50 mg to a daily 62.5 mg dosage achieving further tumor response without any serious adverse events. In August 2009 cerebral progression was confirmed. Then the sunitinib treatment was terminated. After surgical removal of the biggest occipital metastasis, whole brain radiotherapy (RT) was initiated. Having delivered only limited RT dose (4 Gy in 2 fractions), a second neurosurgery had to be carried out due to tumor progression and mass effect. A second-line sorafenib treatment was started in October 2009 and four months later the residual cerebral mass was removed with a third neurosurgical intervention. Up to January 2011 all control examinations showed good tumor regression under continuous sorafenib medication with tolerable (Grade 1–2) side effects. During the year of 2011 due to mediastinal and suprarenal progression sorafenib medication was terminated and without having any effective fourth line systemic treatment first mediastinal irradiation was carried out. This was followed by surgical removal of the suprarenal manifestation. Thus, after achieving excellent tumor control without any systemic therapy, our oncology team recommended the watch and wait approach, and further observation was carried out from the spring of 2012.

**Fig. 2:**
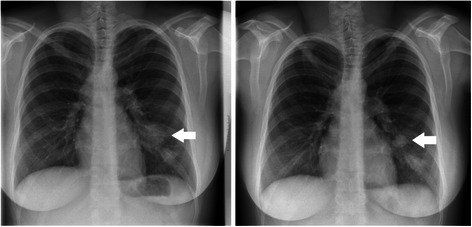
Chest X-ray examination before (left) and after (right) surgical abortion. The diameter of the largest pulmonary manifestation decreased significantly

In August 2012, when the patient was 22 years old, a 13-week-old pregnancy was verified by gynecological examination. During her anticancer treatment she was informed about the importance of using contraception and she reportedly used only physical contraceptive methods. The patient insisted on her pregnancy, although she was fully informed of the risk of her decision. At the end of August the first chest X-ray examination recorded two new pulmonary manifestations, with diameters of 29 × 19 mm and 18 × 11 mm. Twelve days later chest MRI was carried out, showing rapid progression of the lung metastases with largest diameters of 41, 16 and 11 mm, and with bilateral hilar and right supraclavicular lymph node manifestations. Due to the significant progression and the appearance of clinical symptoms our patient changed her decision and accepted the surgical abortion of her pregnancy. During this period the respiratory distress deteriorated. Following the abortion, the patient’s complaints ceased and the size of the neck mass decreased. Chest X-ray examination showed regression compared to the previous X-ray findings (Fig. 2).

**Fig. 3:**
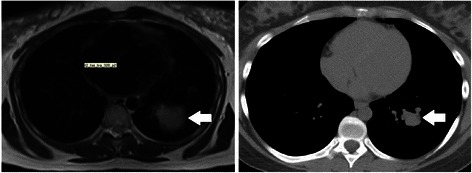
Chest MRI and CT examination before (left) and after (right) surgical abortion. The volume of the largest pulmonary manifestation decreased from 30.02 cm^3^ to 3.51 cm^3^

The patient received ergotamine and bromocriptine medication immediately after the abortion. Eight days after the abortion chest CT examination was carried out to verify the tumor regression. Results revealed rapid regression of the pulmonary manifestations (Fig. 3): the diameter of the largest metastasis decreased to 23 mm and the volume decreased from 30.02 cm^3^ to 3.51 cm^3^ based on the measurements of two independent observers. Without any anticancer treatment, within one week the tumor shrinkage rate was at least 85–90 %, compared to the pre-abortion volume. Meanwhile the diameter of the palpable supraclavicular mass decreased as well, from about 3 cm to 2 cm.

In the beginning of November 2012 the check-up CT showed a stable disease without any therapeutic intervention. One month later the chest X-ray revealed progression in one of pulmonary manifestations, therefore interferon re-induction was initiated based on the hypothetical post-abortion immunological effect and the patient’s preference. In January 2013 restaging CT examinations demonstrated multi-organ progression therefore the interferon medication was terminated. The patient received RT, followed by the fourth neurosurgical intervention in order to remove a frontal cerebral metastasis, which caused mass effect. Completing whole brain RT (36 Gy in 18 fractions) sunitinib re-challenge treatment strategy was introduced in a continuous daily dosing of 25 mg. The next brain MRI showed regression of the residual cerebral lesions however, the whole-body CT revealed unambiguous and rapid abdominal (liver and adrenal gland) and multiple bone progression. The general condition of the patient deteriorated, so we delivered palliative irradiation to the painful vertebral bones (20 Gy in 5 fractions) and medroxy-progesterone medication was initiated. Due to the rapid progression and multi-organ failure, 6 years after the first diagnosis, at the end of June 2013 we lost our patient.

To prove the potential role of hormonal effects during pregnancy IHC examinations were performed on the original biopsy and surgery samples of the primary tumor and the metastases. The presence and density of Estrogen, Progesterone and Luteinizing Hormone Receptors (ER, PGR and LHR, respectively) were analyzed. ER (SP1, rabbit monoclonal, 1:50, Histopathology Ltd.) and PGR (SP2, rabbit monoclonal, 1:100, Histopathology Ltd.) primary antibodies were used (both with Bond Epitope Retrieval solution) and Bond Polymer Refine Detection (Leica, Germany) was applied as a developer system on Bond TM. LHR antibody (H-50: sc-25828, rabbit polyclonal IgG, Santa Cruz Biotechnology, Dallas, Texas) was used as primary antibody at 2ug/ml final concentration with citrate buffered heat retrieval, pH6. Deparaffinized sections were pretreated with EnVision^TM^ FLEX Target Retrieval Solution, 3 in 1, Low pH (20′) - K8005. The reaction was developed on Dako Autostainer (Dako, Denmark) with EnVision^TM^ FLEX, High pH, HRP, Rb/Mo - K800021 according to the vendors’ guideline.

**Fig. 4:**
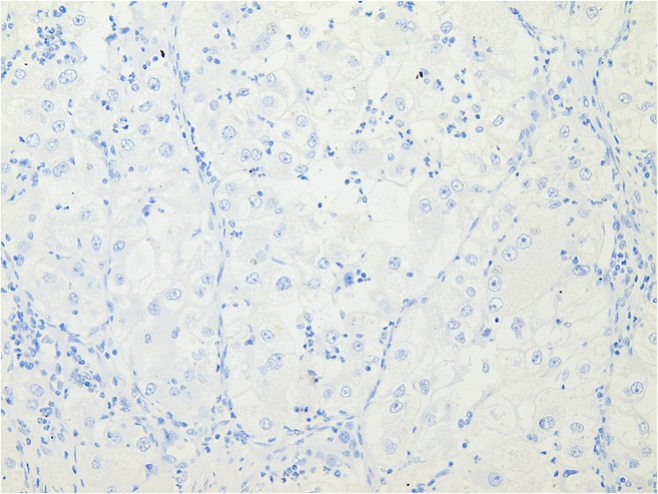
Negative ER status on IHC examination of the tumor

**Fig. 5:**
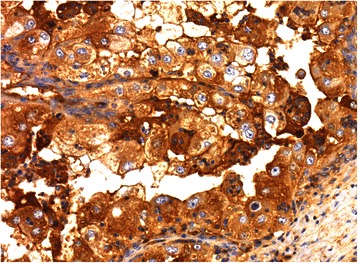
Extremely high density of LHR on IHC examination of the tumor

IHC staining revealed no ER (Fig. 4) or PGR activity. However, a high density of LHR-s was unambiguously detected (Fig. 5), indirectly proving the potential mitogenic effect of human chorionic gonadotropin hormone (hCG).

## Case discussion

Renal cell carcinoma is generally characterized by immunogenic properties and slow progression. The volume doubling time of primary RCC is considered to be about 72 weeks [16]. The progression of mRCC is also generally slow, especially in the elderly [25]. There are several case reports about rapidly growing kidney tumor during pregnancy. For example, Bettez et al. [16] reported a fatal fast growing RCC during pregnancy, the diameter of the renal mass increased from 34 mm to 93 mm in 15 weeks. In the present study a similar growth rate was observed in a few weeks time.

Spontaneous regression of mRCC after surgical intervention or even without nephrectomy is a well-known phenomenon [26]. Sometimes it can be observed in a very fast manner. Otherwise, in the age of targeted therapies, a moderate regression can be realized in the routine clinical practice. We have limited data concerning the speed of the tumor shrinking effect. In the volumetric analyses by Stein the typical shrinkage rate was moderate as well. In case of successful treatments the largest diameter of the tumors came to be halved in 1 to 3 months [27]. In our case a similar shrinkage rate was observed within 8 days. In the routine clinical practice similar rapid tumor reactions can only be observed during the treatment of lymphomas and neuroendocrine small cell carcinomas.

The mechanism of the presently observed enormous tumor shrinkage is not known and may not be expected, moreover, the possible effect of post-abortion medication also cannot be excluded. Nevertheless, several endocrine, immunologic and vascular factors could also play a role in the background. It is widely accepted that some renal cancers express ER and PGR [28]. The dramatic decrease in the serum level of sex hormones due to the surgical abortion could result in an anti-estrogen effect and inhibition of cell division. However we did not succeed in proving any hormonal sensitivity of the present tumor.

Metastatic RCC is considered to be a highly immunogenic tumor. Pregnancy can alter the immune reactions and auto-immunity in case of chronic lymphoid leukemias [11]. Pregnancy is a special immunological state; the hormonal imbalance is important in order to maintain gravidity and the placenta produces several cytokines, tumor necrosis factors, growth and angiogenic factors. Natural killer cells, monocytes can be observed in the decidual tissues thereby influencing the behavior of the malignancies [29, 30]. With the removal of the placenta an inverse immune reaction can be supposed which may inhibit the further growth of the tumor. Nevertheless, in the present study, no significant inflammation or leukocyte, lymphocyte infiltration was noticed in the original tissue sample. However, none of the above hypotheses can elucidate the observed rapid tumor regression, the spectacular necrosis or apoptosis of the cancer cells and the fast elimination of the destroyed tissues.

The angiogenic factors play a key role in the development of renal cell carcinoma. The clinical application of several types of vascular endothelial growth factor (VEGF) inhibitor agents (TKIs as sunitinib, sorafenib, pazopanib or the VEGF ligand binding bevacizumab) dramatically changed the treatment of mRCC [1–3]. The formation of new vessels is an important factor in the development and growth of the placenta as well [31]. Decidual fibroblasts produce different vascular endothelial growth factors. It is also important that the plasma placental growth factor (PlGF) level with angiogenic potential is reportedly higher in mRCC patients. Moreover, PlGF and VEGF level could be associated with the clinical features of RCC [32]. The results of in-vitro mRNA analyses suggest that VEGF, PlGF, and basic fibroblast growth factor work cooperatively to increase the angiogenesis in RCC [33]. Another vascular way could be the rapid change in the level of endocrine gland-derived vascular endothelial growth factor (EG-VEGF). EG-VEGF is an angiogenic factor reported to be specific for the placenta and potentially regulated by hCG [34].

Human chorionic gonadotropin hormone, which is equal to a group of 5 molecules having separate biological functions and often called the “everything molecule”, plays an important role in maintaining decidual functions and pregnancy via angiogenic effect ensuring the growth of the foetus [35]. The role that hCG may play in the oncogenic process of cancer is certainly complex. Nevertheless, it is suspected that hCG is involved in the angiogenesis, in the development of metastasis and the immune escape central to cancer progression. Human chorionic gonadotropin hormone variants antagonize the TGFß receptor, promoting cell growth and blocking cell apoptosis [35–37]. Based on X-ray crystallographic structure studies hCG is considered to be a member of the “cystine knot growth factor/TGFβ (CKGF) oncoprotein superfamily” (TGFβ, PDGFB, VEGF, PlGF, hCG etc.), supposing the cross-talk between the multiple growth regulatory systems [35–37]. HCG stimulates angiogenesis through TGFβ receptor activation, and hCG- TGFβ receptor plays a key role in the angiogenesis associated both with the placental development and the tumorigenesis [38].

It is known that the expression of hCG and its beta subunit is a widespread phenomenon that has been described in many cancer subtypes. The cluster of choriogonadotropin sensitive tumors, such as choriocarcinoma and testicular cancers is well known [36]. The incidence of hCG expression varies in different epithelial tumor types, positive detection ranges from 0 % in RCC to 93 % in small cell lung cancer with an average of 30 % by IHC [37]. Many authors noted the aggressive nature of hCG positive tumors. Moreover hCG expression is more likely to be the result of altered gene regulation and it is regarded as a marker of the presence of pluripotent stem/germ cells [35–37]. In the last decade, several clinical studies tried to prove the therapeutic effect of anti-hCGβ cancer vaccine [35–37]. However, hCG can play a preventive role in breast cancer [37].

As mentioned before in the work of Berzal-Cantaleyo RCC samples showed no hCG positivity by IHC [35]. However, reverse transcription-polymerase chain reaction (RT-PCR) and restriction endonuclease analyses show that 52 % RCC tissue samples proved to be positive for beta hCG mRNA expression [39]. Hotakainen et al. found an increased level of beta subunit in 23–40 % of RCC patients, concluding the negative prognostic value of hCG positivity in their work [40, 41]. Translocation type RCC is generally considered to be a rapidly growing tumor [15]. The aggressive clinical behavior of the tumor in our case is attached to an increased hCG expression, as well. Several other case reports describe hCG producing RCC [42].

The hCG receptor is generally considered to be practically equivalent to luteinizing hormone receptor (LHR) and the examination of LHR overexpression is well accepted in the literature [35, 36]. The role of LHR expression and activation is uncertain in cancer progression, even if it prevents cancer cell proliferation [43]. LHR expression is common in different cancer types, including RCC as well [44]. We analyzed the density of LHR in the original tissue blocks of the patient by IHC and succeeded in proving the potential role of hCG in the course of the disease.

These findings support the theory about the role of placental angiogenic factors and hCG in the growth of a tumor during pregnancy and in the regression after surgical abortion. The enormous shift in vascular activity after abortion could explain the rapid decrease of the tumor mass. Presumably, the rapid decrease in the PlGF and hCG plasma levels may have negative effects on the VEGF plasma levels, as well. The fast tumor shrinkage in such a highly vascularized tumor type as RCC may be explained with the facts above.

## Conclusions

Our case study proved that pregnancy may promote the progression of mRCC. The excess production of hCG which is normally important to maintain gravidity could play a special role in the progression-regression phenomenon of mRCC. However, more new clinical data are necessary to validate the present notions about the general predictive role of LHR overexpression in fast growing cancers during pregnancy. Nevertheless, there is a further need to investigate the effects of angiogenic, growth and endocrine factors. Doing so will help better understand the biological behavior of different RCC types and cancer during pregnancy.

## Consent

Written informed consent was obtained from the relatives of the patient for publication of this case report and any accompanying images. A copy of the written consent is available for review by the Editor-in-Chief of this journal.

## Abbreviations

CK: cytokeratin
EG-VEGF: endocrine gland-derived vascular endothelial growth factor
EMA: epithelial membrane antigen
ER: estrogen receptor
hCG: human chorionic gonadotropin hormone
IHC: immuno-histochemistry
LHR: luteinizing hormone receptor
mRCC: metastatic renal cell carcinoma
PDGFB: platelet-derived growth factor subunit B
PGR: progesterone receptor
PlGF: plasma placental growth factor
RCC: renal cell carcinoma
RT: radiotherapy
TGF: transforming growth factor
TKI: tyrosine kinase inhibitor
VEGF: vascular endothelial growth factor
